# Information Transfer in Gonadotropin-releasing Hormone (GnRH) Signaling: EXTRACELLULAR SIGNAL-REGULATED KINASE (ERK)-MEDIATED FEEDBACK LOOPS CONTROL HORMONE SENSING*

**DOI:** 10.1074/jbc.M115.686964

**Published:** 2015-12-07

**Authors:** Kathryn L. Garner, Rebecca M. Perrett, Margaritis Voliotis, Clive Bowsher, George R. Pope, Thanh Pham, Christopher J. Caunt, Krasimira Tsaneva-Atanasova, Craig A. McArdle

**Affiliations:** From the ‡ Laboratories for Integrative Neuroscience and Endocrinology, School of Clinical Sciences, University of Bristol, Bristol, BS1 3NY, United Kingdom,; § School of Mathematics, University of Bristol, Bristol, BS8 1TW, United Kingdom,; ¶ Texas A and M University Corpus Christi, Corpus Christi, Texas 78412,; ‖ Department of Biology and Biochemistry, University of Bath, Bath, BA2 7AY, United Kingdom, and; ** Department of Mathematics, College of Engineering, Mathematics, and Physical Sciences, University of Exeter, Exeter, EX4 4QF, United Kingdom

**Keywords:** cell signaling, extracellular signal-regulated kinase (ERK), G protein-coupled receptor (GPCR), mathematical modeling, mitogen-activated protein kinase (MAPK), gonadotropin-releasing hormone, mutual information, signal transduction

## Abstract

Cell signaling pathways are noisy communication channels, and statistical measures derived from information theory can be used to quantify the information they transfer. Here we use single cell signaling measures to calculate mutual information as a measure of information transfer via gonadotropin-releasing hormone (GnRH) receptors (GnRHR) to extracellular signal-regulated kinase (ERK) or nuclear factor of activated T-cells (NFAT). This revealed mutual information values <1 bit, implying that individual GnRH-responsive cells cannot unambiguously differentiate even two equally probable input concentrations. Addressing possible mechanisms for mitigation of information loss, we focused on the ERK pathway and developed a stochastic activation model incorporating negative feedback and constitutive activity. Model simulations revealed interplay between fast (min) and slow (min-h) negative feedback loops with maximal information transfer at intermediate feedback levels. Consistent with this, experiments revealed that reducing negative feedback (by expressing catalytically inactive ERK2) and increasing negative feedback (by Egr1-driven expression of dual-specificity phosphatase 5 (DUSP5)) both reduced information transfer from GnRHR to ERK. It was also reduced by blocking protein synthesis (to prevent GnRH from increasing DUSP expression) but did not differ for different GnRHRs that do or do not undergo rapid homologous desensitization. Thus, the first statistical measures of information transfer via these receptors reveals that individual cells are unreliable sensors of GnRH concentration and that this reliability is maximal at intermediate levels of ERK-mediated negative feedback but is not influenced by receptor desensitization.

## Introduction

Most work addressing cell signaling mechanisms involves measurement of aggregate responses from large cell populations despite the fact that this obscures cell-cell variation. Such variation is inevitable, because the underpinning biological processes are stochastic, and is crucial for the behavior of cell populations (1) in which each individual cell must sense the environment and make appropriate fate decisions (to express or suppress given genes, to survive, die, proliferate, or differentiate). Information theory was developed to analyze electronic communication but can also be applied to biological systems, where it provides tools with which the influence of cell-cell variation on the reliability of sensing can be determined (1–8). In this context information is defined as the uncertainty about the environment that is reduced by signaling and can be quantified as the mutual information (MI)^3^ between two stochastic variables such as signal and response in a biological pathway (1). MI measures the quality of the inference (or “prediction”) of the signal from the response, does not require knowledge of the transduction mechanism and is unaffected by non-linear transformations of the signal or response (1).

This approach takes variability into account rather than just considering the average response, and its merit can be illustrated by consideration of a multi-tiered signal transduction pathway. Conventional wisdom is that signal amplification occurs through the cascade, but in reality information about the signal cannot actually increase from one tier to the next so any increase in numbers of activated molecules must be associated with increased variability (noise). In fact, there is most likely loss (and never gain) of information through such cascades, raising the question of how cells mitigate the loss. Here, negative feedback loops are of particular interest as they could reduce information transfer (by reducing dynamic range of the output) or protect it (by reducing cell-cell variability). Indeed, work with tumor necrosis factor signaling to NFκB and ATF-2 revealed that negative feedback increased information transfer with 30 min of stimulation but decreased it at 4 h (2). The extracellular signal-regulated kinase (ERK, (we use the term ERK to mean ERK1 and/or ERK2, giving the numerical designation when a specific form is meant) cascade is of particular interest in this regard as ERK responses are modulated by feedback loops that shape population-averaged ERK responses and influence cell fate (9–11). Distinct feedback loops operate on different time scales with ERK-mediated inhibitory phosphorylation of Raf or son-of-sevenless (SOS) occurring rapidly (5–15 min), whereas ERK-driven expression of dual specificity phosphatases (DUSPs) (12) has time delays for transcription and translation (>40 min). Exploring the reliability of EGF sensing, we recently showed that ERK-mediated negative feedback could increase information transfer by reducing constitutive (*i.e.* basal) ERK activation (4).

Cells receive information via GPCRs that signal via G-proteins to specific effectors and are major targets for drug therapy. Desensitization of GPCR-mediated responses is often thought of as a means of protecting cells from over-stimulation, but to our knowledge information theory has not been applied to measure precisely how such adaptive measures (including negative feedback loops shaping ERK signaling) actually influence sensing via GPCRs. Here we do so using gonadotropin-releasing hormone receptor (GnRHR) signaling as a model system. GnRHR are G_q/11_-coupled GPCRs in the pituitary that mediate central control of reproduction (13). When activated by the neuropeptide GnRH, they cause a phospholipase C (PLC)-mediated increase in the cytoplasmic Ca^2+^ concentration that drives exocytotic gonadotropin secretion. This Ca^2+^ elevation also has marked effects on transcription, in part mediated by the Ca^2+^/calmodulin-mediated activation of NFAT (nuclear factor of activated T-cells) (13, 14). GnRHR-mediated PLC activation also activates protein kinase C (PKC) isozymes and causes a (largely) PKC-mediated ERK activation (13, 15). ERK then mediates effects of GnRH on gene expression (13, 16–21) including increased expression of nuclear-inducible DUSPs (13, 20, 21). These, in addition to more rapid ERK-mediated negative feedback loops, modulate ERK responses to GnRH (20). GPCR stimulation typically provokes receptor desensitization within seconds to minutes (22). The active receptor is typically phosphorylated within its COOH-terminal intracellular tail by GPCR kinases, and this facilitates binding of arrestins that inhibit G-protein activation. However, GnRHR have undergone a period of accelerated molecular evolution with the advent of mammalian GnRH being associated with the loss of COOH-terminal tails (13, 23). Most mammalian GnRHRs, therefore, do not show agonist-induced phosphorylation, arrestin binding, and rapid homologous desensitization, whereas all non-mammalian GnRHRs characterized to date do (13, 17, 23–26). Accordingly, GPCR signaling to ERK can be treated as a noisy communication pathway with the potential for negative feedback occurring rapidly (ERK-mediated phosphorylation of Raf, within 5–15 min), slowly (DUSP mediated inactivation of ERK (beyond 40 min), or via an ultrafast-feedback loop (receptor desensitization, within seconds to minutes). We can avoid the latter by using mouse (m) or human (h) GnRHR or engage it by using *Xenopus laevis* (X) GnRHR.

Here we use MI to quantify information transfer via GnRHR and show that these values are low (<1 bit) for GnRHR signaling to ERK and NFAT irrespective of whether acute or transcriptional responses are monitored and for a heterologous expression system (adenovirus (Ad) GnRHR-transduced HeLa cells) as well as for native GnRHR in LβT2 gonadotropes. We developed a stochastic model for ERK activation with fast and slow negative feedback loops which predicts that both have the potential to harm or protect sensing and confirm this by manipulating ERK-dependent feedback experimentally. We also show that GnRH sensing via mGnRHR or hGnRHR is comparable to that via XGnRHR or h.XGnRHR (hGnRHR with the COOH-terminal tail added from XGnRHR). Thus we consider three negative feedback loops and show that two of them (the distal ERK-mediated loops) do indeed influence information transfer through the ERK pathway. Most importantly, we find that processes causing “desensitization” of ERK responses can increase the reliability of GPCR-mediated sensing.

## Experimental Procedures

### 

#### 

##### Cell Culture and Transfection

HeLa cells (from ECACC) were cultured in DMEM with 10% fetal calf serum (FCS) as described (27, 28). They were trypsinized and seeded at 3–5 × 10^3^ cells/well in Costar black-walled 96-well plates (Corning, Arlington, UK). After ∼16 h they were incubated 4–6 h in DMEM, 2% FCS, and Ad expressing mGnRHR, hGnRHR, XGnRHR, a signal dead point mutant of the hGnRHR (A261K.hGnRHR) or a chimera of the hGnRHR with the COOH-terminal tail from the XGnRHR (h.XGnRHR) (20, 29). For a transcriptional read-out of ERK activity, cells received Ad for an Egr1 promoter driving expression of zsGREEN (Ad Egr1-zsGREEN) (28). For DUSP5 experiments cells were transduced with Ad Egr1 DUSP5 myc (myc-tagged DUSP5 expression driven by the Egr1 promoter) or a mutant with reduced affinity for ERK (Ad Egr1 R53A/R54A DUSP5 myc) (30). Medium was then replaced with DMEM, 0.1% FCS, and the cells were incubated ∼16 h before stimulation (21). For some experiments ERK was knocked-down by transfection with two siRNA duplexes each for ERK1 and ERK2 as described (27). Where ERK was knocked down, Ads were used to add back previously characterized (31) reporters consisting of wild-type (WT) ERK2 in tandem with GFP (ERK2-GFP) or a catalytically inactive mutant (K52R ERK2-GFP). In some experiments cells received Ad to express an NFAT1c-EFP translocation reporter or a transcriptional reporter consisting of an NFAT-response element (NFAT-RE) driving expression of asRED at the same time as the Ad GnRHR (14, 28, 32). Ads were used at 1–10 plaque-forming units (pfu)/nl, except where they were varied over a broader range as detailed in the legends. Where multiple HA-GnRHR were compared, the transduction protocol was modified to provide a broad range of expression levels as described under Figs. 9 and 10. For some experiments the gonadotrope-derived LβT2 cell line was used. These were kindly provided by Prof. P. L. Mellon (University of California, San Diego, CA). They were cultured as described (14) and transduced with Ad for reporter expression as above. These cells have endogenous mGnRHR and were, therefore, not transduced with Ad GnRHR. The stimuli used (at concentrations shown in figure legends) were GnRH, GnRH II, or phorbol 12,13-dibutyrate (PDBu) and ionomycin (each from Sigma).

##### High Content Imaging and Data Analysis

After stimulation cells were fixed and analyzed as described (33). For some experiments ERK2-GFP, NFAT1c-EFP, zsGREEN, or asRED were also visualized. Image acquisition was automated using an InCell Analyzer 1000 (GE Healthcare) with a 10× objective and filters for DAPI (blue channel), Alexa488, GFP, EFP, and zsGREEN (green channel), or Alexa546 and asRED (red channel). Image analysis was as described (33), determining whole cell or nuclear fluorophore intensities in arbitrary fluorescence units (AFU). Replicate treatments in 2–4 wells of cultured cells were pooled to produce population-averaged responses that were pooled from multiple experiments (28, 31, 33). For most experiments we constructed full concentration response curves (*i.e.* control and 10^−12^–10^−6^ m GnRH) at multiple time points (5 min–6 h) and collected images for 4–9 fields of view per well. This yielded data for >10,000 individual cells (for each treatment in each experiment). These individual cell measures were used to calculate MI between stimulus concentration and the experimental readouts at each time point. For one series of experiments we also calculated MI between measured HA-GnRHR and ppERK levels. The analysis was performed in MatLab (MathWorks, Natick, MA) using the equation,

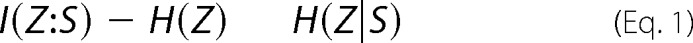
 where *I* is the mutual information between a signal (*S*) and a response (*Z*), *H*(*Z*) is the unconditional entropy of the response, and *H*(*Z* |*S*) is the conditional entropy (4).

##### Stochastic Simulations

We modeled ERK signaling as a dynamical system with two negative feedback loops: a fast one from ppERK to effectors E and E* and a slow one via phosphatase (p-ASE_2_) expression (Fig. 3, see Table 1 for parameters). The Gillespie algorithm was used for stochastic simulations to study the behavior of the model at different feedback strengths. Fast negative feedback was controlled by varying the binding rate of ppERK to E or E* and slow negative feedback by varying the number of promoters expressing p-ASE_2_. MI was estimated from 2000 independent runs of the model with total ERK sampled from a log_10_ normal distribution, with a mean 3.9 and S.D. 0.1. The model was run to steady state before the signal was introduced. The number of signal molecules (*S*) was randomly drawn from a uniform distribution on the logarithmic scale between 10 and 1000 molecules.

## Results

### 

#### 

##### Using MI to Measure Information Transfer via GnRHR to ERK

**FIGURE 1.**
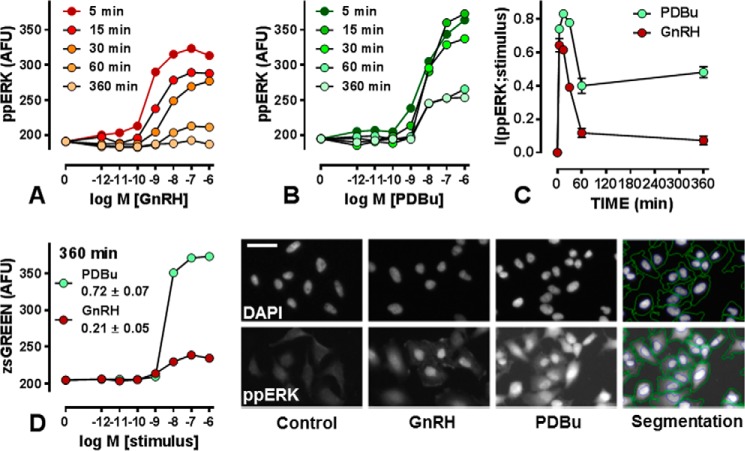
**Quantifying GnRHR-mediated ERK signaling in HeLa cells.** *Panels A* and *B* show population-averaged nuclear ppERK in HeLa cells transduced with Ad-mGnRHR and Ad-Egr1-zsGREEN and stimulated 5, 15, 30, 60, or 360 min with 0 or 10^−12^–10^−6^ m GnRH as indicated. Cells were fixed and stained (DAPI and ppERK) before image capture and analysis. The data are the means (*n* = 3) from three separate experiments, each with triplicate wells and 3 fields of view/well with S.E. omitted for clarity. Background values (without fluorophore) were 120–150 AFU and were not subtracted. Individual cell measures underlying the data in *A* and *B* were used to calculate (I(ppERK;stimulus)) and are shown in *panel C* (mean ± S.E., *n* = 3). *Panel D* shows population-averaged zsGREEN values for cells stimulated for 6 h with the indicated concentration of GnRH or PDBu, and the corresponding I(Egr1;stimulus) values are shown (mean ± S.E., *n* = 3). The microscope images show representative DAPI- and ppERK-stained cells cultured under control conditions or stimulated 5 min with 10^−7^ m GnRH or PDBu as indicated. The *horizontal bar* is ∼20 μm, and the *right hand images* show an example of the automated image segmentation used to define perimeters of the nuclei and cells (perimeters superimposed over PDBu-treated cells). Each image shows <1% of the area captured to generate the *x-y* plots.

GnRHR-expressing HeLa cells were stimulated for varied periods with GnRH before staining and imaging. Representative images are shown in Fig. 1, and image analysis revealed that GnRH caused concentration-dependent increases in ppERK that were rapid (maximal at 5 min) and transient (near basal at 60–360 min) (Fig. 1*A*). The population-averaged data shown are derived from >10^6^ cells, and these single cell measures were used to calculate MI between GnRH and ppERK. As shown (Fig. 1*C*), I(ppERK;GnRH) increased rapidly to >0.6 bits at 5 min with a gradual reduction to <0.2 bits by 60–360 min. Similarly, the PKC activator (PDBu) caused dose-dependent increases in ppERK, but these were slower and more sustained (maximal at 5–30 min and substantially above basal even at 360 min, Fig. 1*B*). I(ppERK;PDBu) increased rapidly to >0.8 bits between 5 and 30 min and then reduced to ∼0.4 bits at 60 and 360 min (Fig. 1*C*). The cells were also transduced with recombinant Ad expressing an Egr1 promoter driving zsGREEN expression (Ad-Egr1-zsGREEN) for an imaging readout of ERK activation (28). Neither stimulus increased zsGREEN levels at 5–60 min (not shown), whereas both caused dose-dependent increases at 360 min (Fig. 1*D*). The maximal PDBu effect was greater than that of GnRH because sustained ERK activation drives ERK-mediated transcription more effectively than transient ERK activation (28). Similarly, I(Egr1;stimulus) was greater for PDBu (0.72 ± 0.07) than for GnRH (0.21 ± 0.05). Together, these data revealed that MI can be used to measure information transfer via GnRHR to ERK and that for each input, output, and time point considered, the MI values approximately paralleled the dynamic range observed for the population-averaged responses.

##### Dependence on Effector, Context, and Receptor Number

**FIGURE 2.**
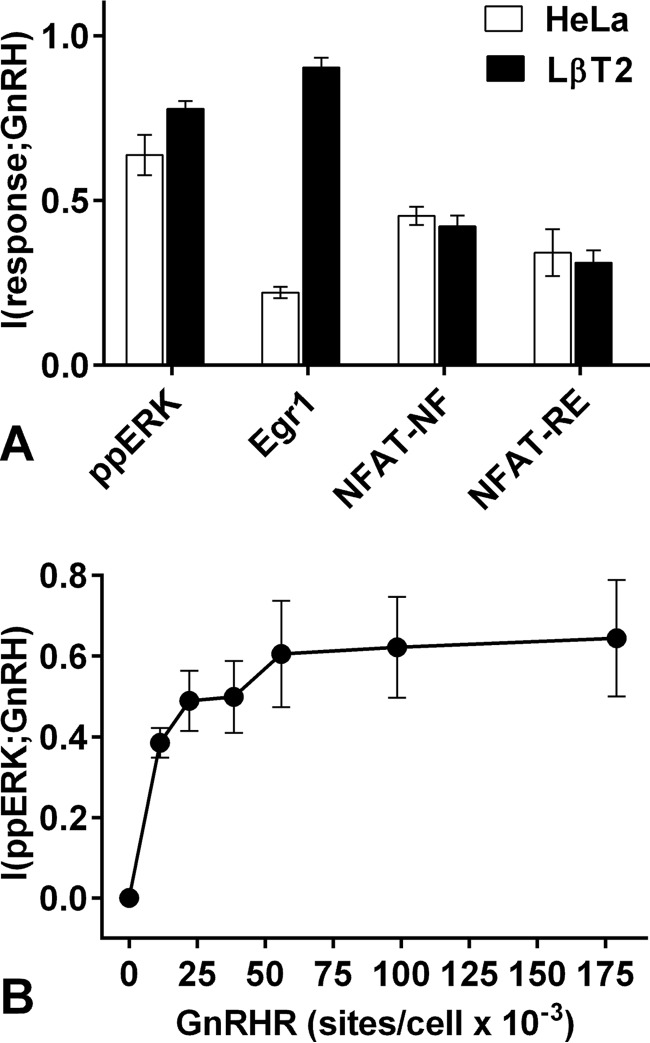
**Quantifying GnRHR-mediated ERK and NFAT signaling in HeLa and LβT2 cells.** *Panel A*, HeLa cells (*open bars*) were transduced with Ad mGnRHR and Ad Egr1 zsGREEN and treated with 0 or 10^−12^–10^−6^ m GnRH before staining, imaging, and calculation of I(ppERK;GnRH) from 5-min data and I(Egr1;GnRH) from 360-min data as described under Fig. 1. Alternatively, they were treated with Ad m-GnRHR and Ad NFAT1c-EFP and Ad NFAT-RE asRED and stimulated with 0 or 10^−12^–10^−6^ m GnRH before staining and imaging. Whole cells asRED and the nuclear fraction of NFAT1c-EFP (NFAT-NF) were calculated for each individual cell, and these values were used to calculate I(NFAT-NF;GnRH) and I(NFAT-RE;GnRH) using 20- and 360-min data, respectively. Parallel experiments were performed with LβT2 cells (*filled bars*) with identical procedures except that the LβT2 cells express endogenous GnRHR and, therefore, did not receive Ad-mGnRHR. The I(response;GnRH) values shown are the means (±S.E., *n* = 3). *Panel B*, HeLa cells transduced with Ad-mGnRHR were stimulated with 0 or 10^−12^–10^−6^ m GnRH and processed as described above except that Ad m-GnRHR titer was varied (0, 0.325, 0.625, 1.25, 2.5, 5, and 10 pfu/nl). The I(ppERK;GnRH) values shown are the means (±S.E., *n* = 3) and are plotted against GnRHR number estimated as described under “Experimental Procedures.”

The relatively low MI values in Fig. 1 imply that the individual cells do not reliably sense GnRH (see “Discussion”) so we considered the possibility that this is a specific feature of our heterologous GnRHR expression system. To address this we compared signaling in HeLa cells (treated with Ad GnRHR and Ad Egr1-zsGREEN and stimulated 5 min for ppERK measures and 6 h for zsGREEN) and LβT2 cells. The latter are a mouse gonadotrope-derived cell with endogenous GnRHR and, therefore, did not receive Ad GnRHR. ppERK responses to GnRH are more sustained in LβT2 cells than in HeLa cells, but the initial effects are comparable (20), and consistent with this, we found that 5 min with 10^−7^ m GnRH increased nuclear ppERK by 100 and 108 AFU in LβT2 and HeLa cells, respectively (data not shown). I(ppERK;GnRH) values (at 5 min) were also similar for both models (Fig. 2*A*), whereas I(Egr1;GnRH) was greater for LβT2 cells than in the HeLa model (0.90 ± 0.03 and 0.22 ± 0.02, respectively). This is presumably because the more sustained ppERK response in LβT2 cells drives a greater transcriptional response, and in accord with this, we found that Egr1-zsGREEN response to 10^−7^ m GnRH was ∼8-fold greater in LβT2 cells than in HeLa cells (not shown). Thus, more sustained activation of ERK is associated with more reliable hormone sensing as determined using the transcriptional readout, but most importantly, MI values were <1 for both cell types and for both ERK activation measures.

It is also possible that sensing is more reliable through the PLC/Ca^2+^/calmodulin branch of the GnRH network than through the PLC/PKC/ERK pathway so we addressed this using two readouts for activation of the Ca^2+^/calmodulin-sensitive transcription factor NFAT. GnRH causes nuclear translocation of an NFAT1c-EFP reporter that is maximal at 20–60 min and can be quantified by measuring the nuclear fraction of NFAT1c-EFP (NFAT-NF) (14). It also increases NFAT-driven transcription that is maximal at 4–8 h as seen with a reporter with the NFAT response element driving asRED expression (NFAT-RE). Kinetics of these responses are similar in HeLa and LβT2 cells (Ref. 14 and data not shown) so we transduced HeLa cells with Ad for GnRHR and the reporters or LβT2 cells with Ad for the reporters alone, and constructed dose-response curves for GnRH (20 min stimulation for NFAT1c-EFP and 6 h for NFAT-RE asRED). I(NFAT-NF;GnRH) values were similar (0.45 ± 0.03 and 0.42 ± 0.02 bits in HeLa and LβT2 cells, respectively) and were lower than the I(ppERK;GnRH) values (Fig. 2*A*). I(NFAT-RE;GnRH) values were also relatively low and similar (0.34 ± 0.07 and 0.31 ± 0.04 bits in HeLa and LβT2 cells, respectively).

We also tested the relevance of GnRHR numbers by transducing HeLa cells with Ad mGnRHR at varied titers (0 or 0.8–25 pfu/nl). From earlier radioligand binding (13, 29, 34, 35) we estimate that this yields 0 or ∼20–180 × 10^3^ GnRHR per cell. Dose-response curves revealed the expected Ad titer-dependent effect of GnRH on ppERK (5 min of stimulation with 10^−7^ m GnRH increased nuclear ppERK values by 74, 105, 119, 146, 175, and 188 AFU after Ad mGnRHR at 0.8, 1.6, 3.2, 6.25, 12.5, and 25 pfu/nl, respectively). The single cell data from these curves (not shown) was used to calculate I(ppERK;GnRH), and this increased from ∼0.25 bits at the 0.8 pfu/nl to ∼0.6 bits at 25 pfu/nl (Fig. 2*B*). Mouse gonadotropes and gonadotrope-derived cell lines express ∼25–75 × 10^3^ GnRHR (13, 29, 34, 35), so this strategy enables control of expression through a range encompassing physiological levels. Together these data suggest that GnRH sensing is indeed dependent upon GnRHR number within a physiologically relevant range and that the Ad mGnRHR titer routinely used herein provides near maximal sensing at a physiologically meaningful GnRHR level.

##### Stochastic Simulations of Information Transfer to ERK

**FIGURE 3.**
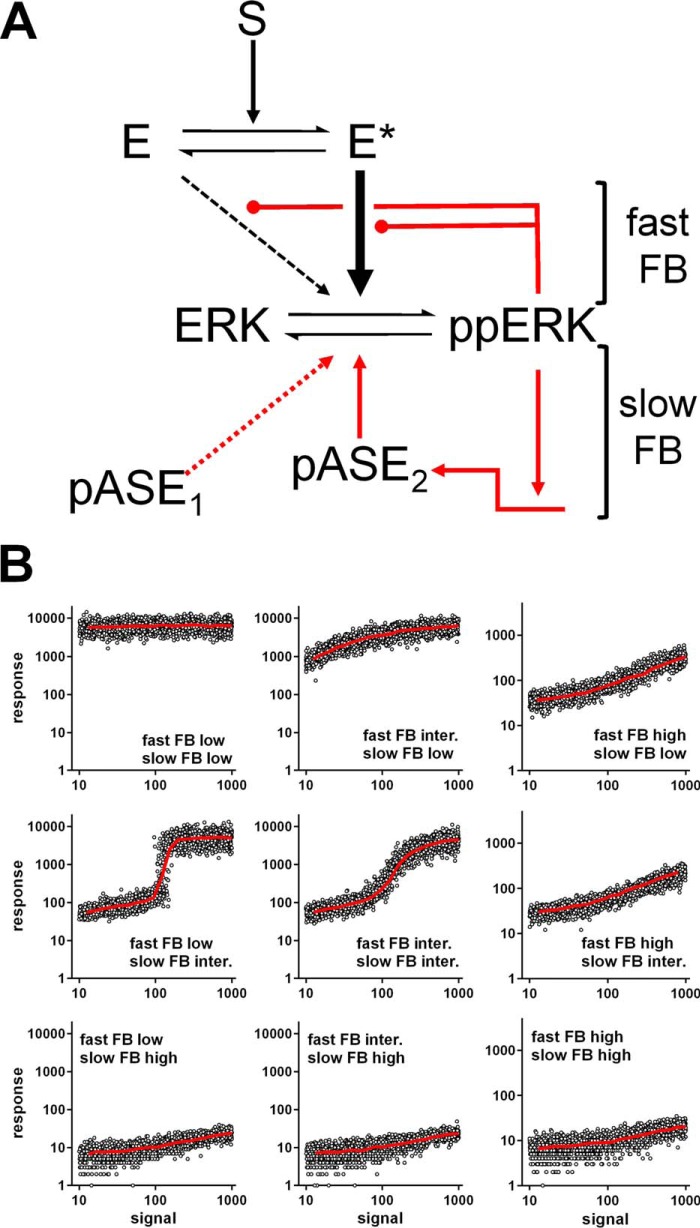
**Stochastic modeling of signaling to ERK.** *Panel A*, ERK signaling model incorporating two negative feedback loops: a fast one from ppERK to effector E and a slow one via phosphatase (*p-ASE*_2_) expression. *Panel B*, we ran stochastic simulations of this model using the parameters in Table 1, varying the strength of the fast and slow negative feedback (*FB*) loops and for each condition using 2000 independent runs of the model. For each run we sampled the total ERK levels from a log_10_ normal distribution (mean of 3.9 and a S.D. 0.1) reaching steady state before signal introduction. Signal molecule number was randomly drawn from a uniform distribution on the log_10_ scale between 10 and 1000 molecules, and for all simulations the stochastic simulation (Gillespie) algorithm was used, as described under “Experimental Procedures.” The figure shows data for 60 min stimulations with fast FB set at low, intermediate, or high strength (0.01, 1.66, and 330, respectively), and slow FB was set at low, intermediate, and high strength (1, 100, and 10000, respectively) with the line of best fit (*red*) superimposed over individual run values (*open circles*). These are from a larger series of simulations run for 5–360 min with a wider range of fast FB and slow FB values, from which the MI values shown in Fig. 4 and Fig. 12 were calculated.

**FIGURE 4.**
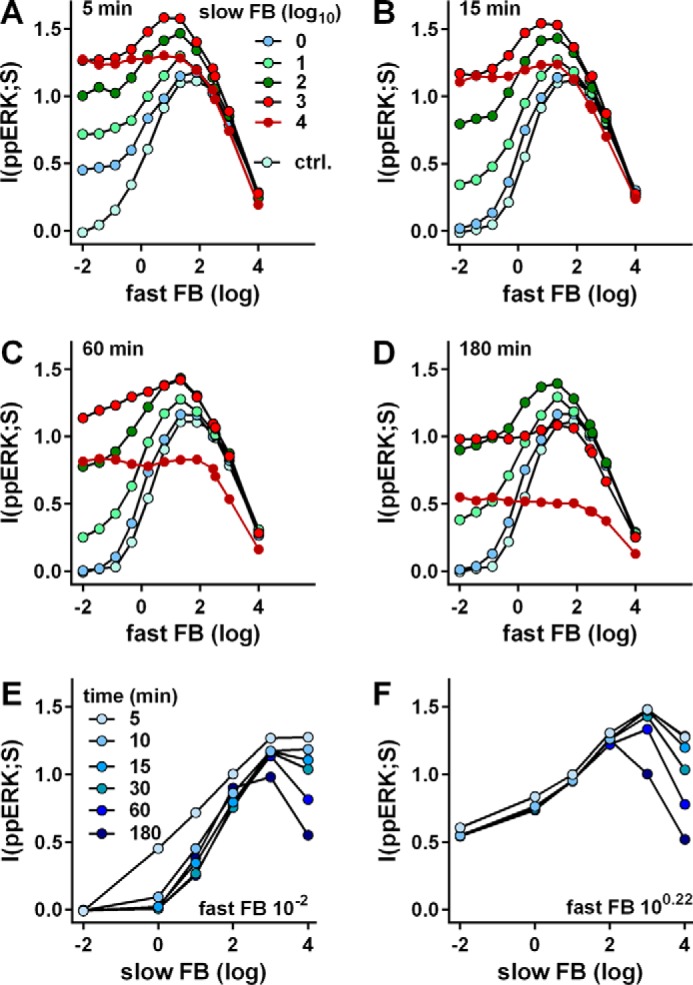
**Stochastic modeling of signaling to ERK.** Model simulations were run as described in the Fig. 2 legend but with a larger range of feedback (*FB*) strengths that were varied as described under “Experimental Procedures” (*i.e.* fast negative feedback was controlled by varying the binding rate of ppERK to E or E* and slow negative feedback by varying the number of promoters expressing p-ASE_2_, and both are shown on log_10_ scales). Information transfer (*vertical axes*) was estimated as MI between signal (*S*) and ppERK at different time points. For panels *A–D* the *x* axis is log_10_ fast FB strength, and the legend shows log_10_ slow FB strength. Also shown is the control (*ctrl.*) with no slow FB. For *panels E* and *F* the *x* axis is log_10_ slow FB strength, and MI values are shown at the indicated stimulation period (5–180 min) with very little fast FB (10^−2^, *panel E*) or with intermediate fast FB (10^0.22^, *panel F*). These are replotted from *A–C* with additional data from 10 and 30 min stimulations.

Having established that MI values for GnRH sensing are relatively low, we explored features that could mitigate information loss, focusing on ERK signaling. ERK is influenced by feedback loops with distinct time frames and mechanisms, and we have described a stochastic model for phosphorylation and dephosphorylation incorporating leak (*i.e.* constitutive activation) and negative feedback. Simulations revealed that negative feedback could protect sensing by inhibiting leak, and this was confirmed for ErbB signaling (4). Here, we adapt this approach to a system (Fig. 3*A*) in which a stimulus (signal (*S*)) causes conversion of an upstream effector (E) into a more active form (E*), that in turn promotes phosphorylation of ERK to ppERK. Constitutive activity is introduced by allowing E to activate ERK (albeit at a much lower rate than E*). We also incorporate ERK-mediated feedback; a fast loop with ppERK inhibiting its activation by E or E*, and a slow one with ppERK driving expression of phosphatase (p-ASE) that inactivates ppERK by dephosphorylation (see the parameters in Table 1). We impose constant leak and a Gaussian distribution on ERK concentration but vary strength of the feedback loops. The model is run without stimulation (S = 0) to steady state and then subjected to uniformly distributed stimulus concentrations for varied duration. Representative model simulation results are given in Fig. 3*B*, and the corresponding MI values for a larger series of simulations are in Fig. 4. The simulations reveal bell-shaped relationships between fast feedback intensity and I(ppERK:S) at all time points, and this is particularly evident when slow feedback is negligible (*blue curves* in Fig. 4, *A–D*). Here, sensing is greatest at intermediate levels because strong fast feedback reduces the response dynamic range, whereas with weak fast feedback the constitutive activity of E generates noise that harms sensing. Conversely when fast feedback is very low, increasing slow feedback increases MI by opposing constitutive ERK activation during pre-equilibration (when S = 0). This is evident for fast feedback of 10^−2^ in Fig. 4*A* (5 min data), and the data for low fast feedback (10^−2^) with varied slow feedback are re-plotted in Fig. 4*E* to illustrate the fact that increasing slow feedback protects sensing initially (5 min) but that a non-monotonic relationship develops over time. This effect is even more prevalent with an intermediate level of fast feedback (10^0.22^ in Fig. 4*F*) where I(ppERK;S) is always maximal at intermediate levels of slow feedback. Thus the simulations predict sensing to be dependent on the complex interplay of fast and slow feedback, but for broad ranges of feedback strength, that information transfer is greatest at intermediate levels of fast and slow feedback.

**TABLE 1:**
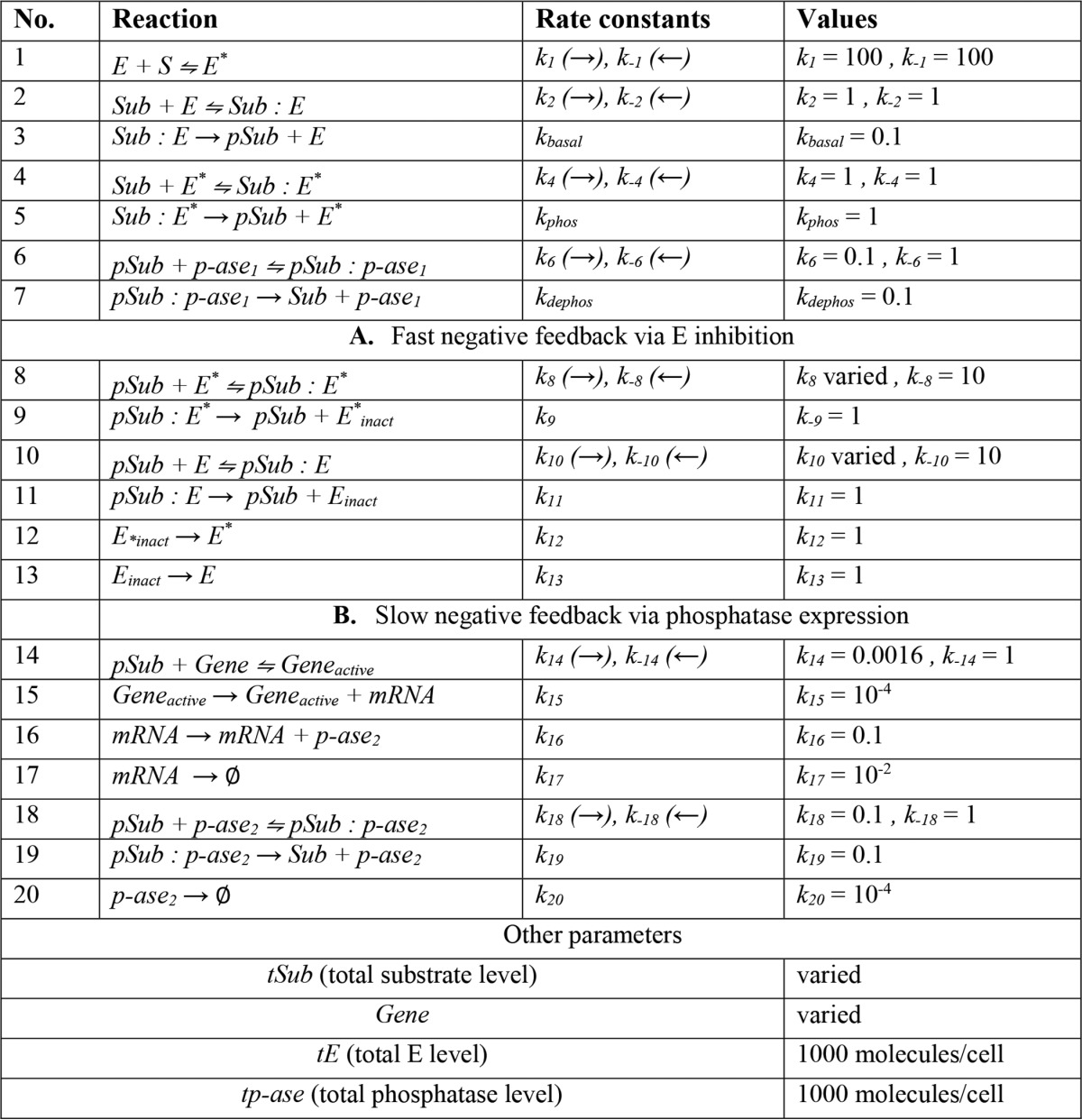
**Parameters used for stochastic modeling of ERK activation** All reactions are formulated in terms of stochastic mass action kinetics. First order rate constants are given in [molecules s]^−1^, and second order rate constants are in [molecules]^−2^ [s]^−1^. *S* is signal, *E* is kinase, *Sub* is substrate, *p-ase*_1_ and *p-ase*_2_ are phosphatases. *Log*_10_*Sub* is approximately normally distributed with mean 3.9 and variance 0.1. When using this approach to model ERK activation (as in Figs. 2, 3, and 9), substrate (*Sub*) and phosphorylated substrate (*pSub*) are equivalent to ERK and ppERK, respectively.

##### Catalytic ERK Activity Supports GnRHR-mediated Information Transfer

**FIGURE 5.**
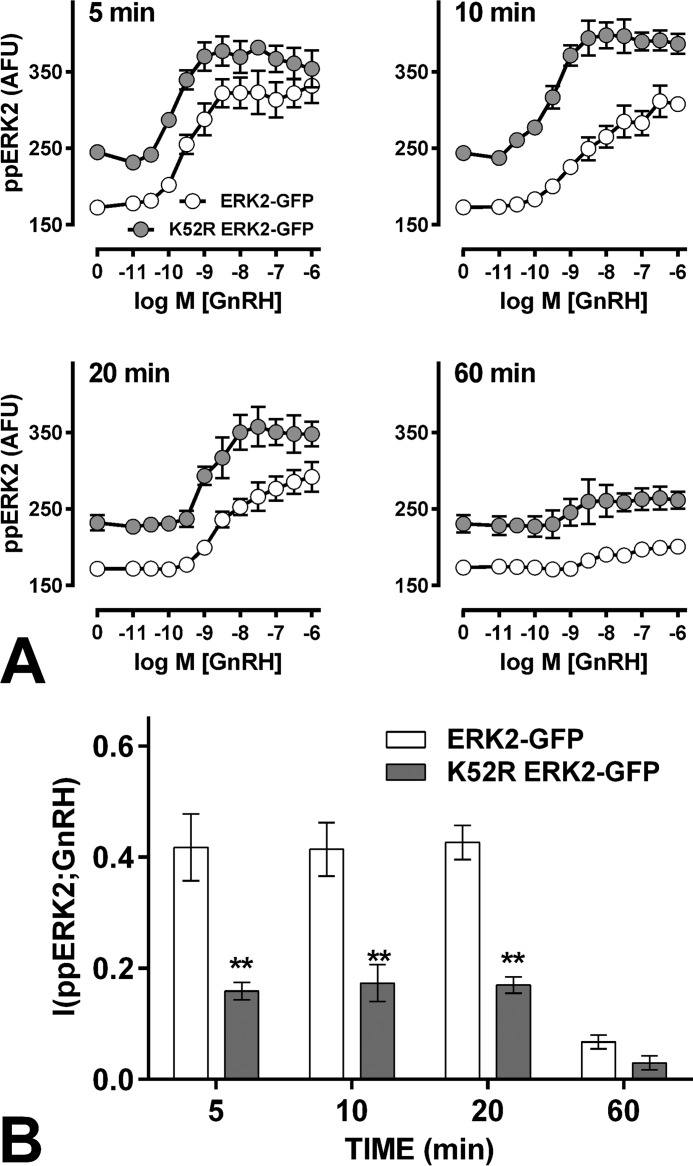
**Inhibition of ERK-mediated feedback impairs GnRH sensing.** *Panel A* shows population-averaged ppERK2 levels after knockdown of endogenous ERK and add-back with Ad ERK2-GFP or Ad K52R ERK2-GFP before stimulation for 5, 10, 20, or 60 min with GnRH (0 or 10^−12^–10^−6^ m). Cells were fixed and stained (DAPI and ppERK), and the single cell ppERK measures were used to calculate the I(ppERK2;GnRH) values in *panel B*. The data are from three separate experiments each with triplicate wells and three fields of view per well. Two way ANOVAs of the ppERK2 measures (*A*) revealed significant differences between ERK2- and K52R ERK2-expressing cells (at all times). Similar ANOVAs of the MI measures (*B*) revealed a significant difference between ERK2- and K52R ERK2-expressing cells (*p* < 0.01) and post hoc Bonferroni tests revealed significant differences at 5, 10, and 20 min (**, *p* < 0.01). These data are from a series of experiments performed in parallel with those shown in Fig. 1.

To manipulate ERK-mediated feedback we first knocked-down endogenous ERKs and used Ad to add-back ERK2-GFP at a similar level to endogenous ERKs (21, 27). Alternatively, catalytically inactive K52R ERK2-GFP was expressed to compare feedback-broken and feedback-intact cells (21, 27). Population-averaged ppERK responses to GnRH were similar in these cells (Fig. 5*A*) and in cells with endogenous ERKs (Fig. 1*A*) with GnRH causing dose-dependent increases in ppERK that were maximal at 5 min and near basal by 60 min. Expressing catalytically inactive ERK2 increased basal ppERK levels (Fig. 5*A*), demonstrating constitutive activation that is normally offset by negative feedback. GnRH also caused a dose-dependent increase in population-averaged ppERK, with the GnRH and K52R ERK2 effects being approximately additive (Fig. 5*A*). Most importantly, I(ppERK;GnRH) was greater in ERK2-expressing cells than in K52R ERK2-expressing cells (at 5, 15, and 60 min), revealing that ERK catalytic activity improves GnRH sensing (Fig. 5*B*), presumably because it mediates negative feedback.

##### Increasing ERK-mediated Feedback Reduces GnRHR-mediated Information Transfer

**FIGURE 6.**
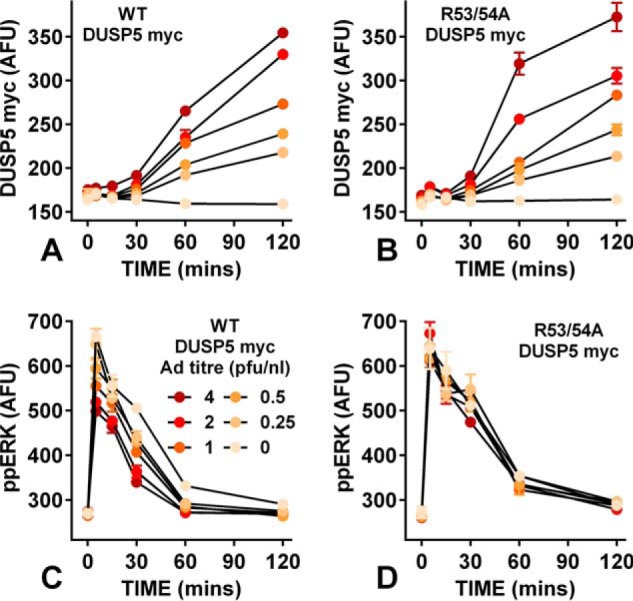
**Influence of DUSP5 induction on GnRHR-mediated ERK signaling.** Cells were treated as described under Fig. 1, except that they were transduced with Ad Egr1 DUSP5-myc or with Ad Egr1 R53A/R54A DUSP5-myc at 0 or 0.25–4 pfu/nl (as indicated) as well as Ad mGnRHR. They were stimulated for the indicated times with 10^−7^ m GnRH and were stained for nuclei (DAPI), ppERK, and myc. The data shown are from a single representative experiment with 2 wells per treatment. *Panels A* and *B* show nuclear DUSP5-myc, whereas *panels C* and *D* show nuclear ppERK levels (mean ± S.E. (*n* = 2)) in AFU, and the legend in *panel C* (Ad titers in pfu/nl) applies to all four panels. Note that the ppERK response to GnRH were reduced at all Ad DUSP5-myc titers (*panel C*) but not by any Ad R52/53A DUSP5-myc titer (*panel D*).

**FIGURE 7.**
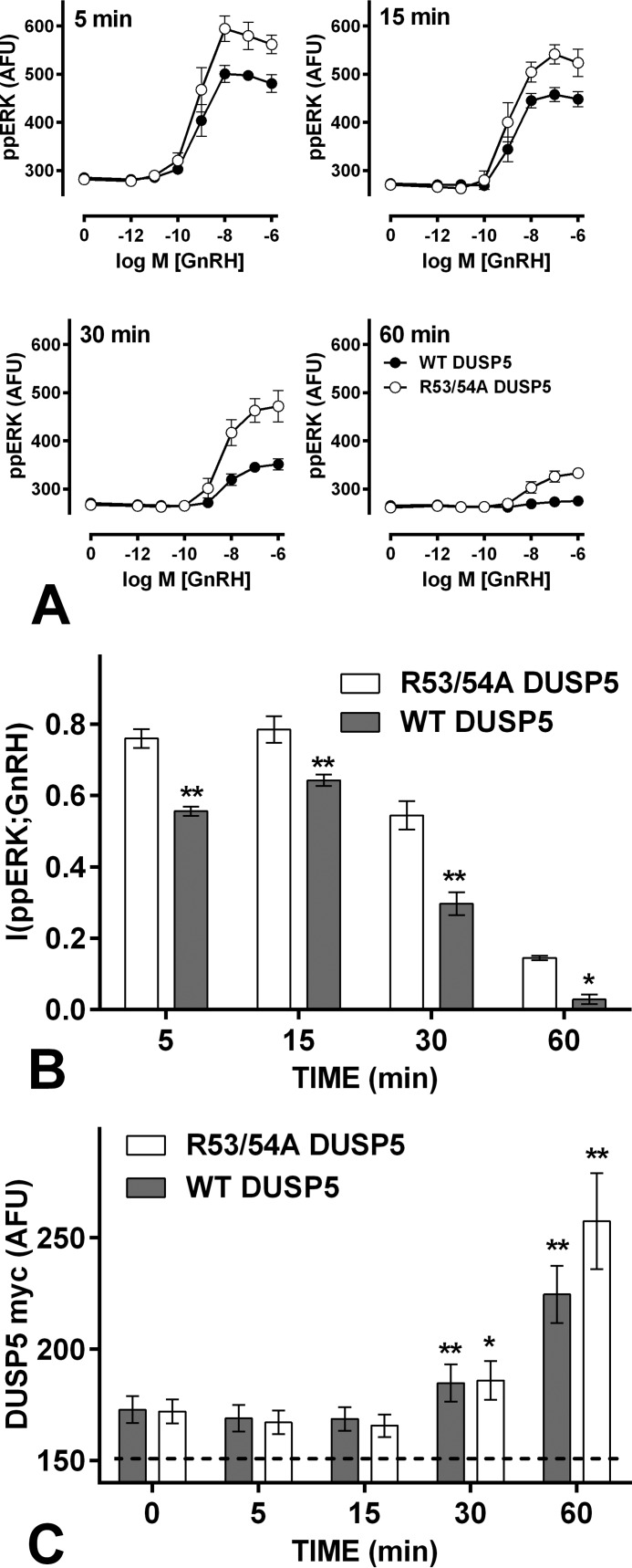
**Increasing ERK-mediated feedback impairs GnRH sensing.** *Panel A*, data from cells treated and imaged as in Fig. 1, except that they were transduced with Ad WT DUSP5myc (*filled circles*) or Ad R53A/R54A DUSP5 myc (*open circles*) at 4 pfu/nl. The data are pooled from three separate experiments, each with triplicate wells and at least three fields of view per well (mean ± S.E., *n* = 3). *Panel B*, corresponding I(ppERK;GnRH) values (mean ± S.E., *n* = 3). *Panel C*, corresponding myc expression levels in cells receiving 10^−7^ m GnRH (mean ± S.E., *n* = 3, the *dashed line* shows the background stain intensity). *A*, two way ANOVAs of the ppERK measures revealed significant differences between WT DUSP5 myc- and R53A/R54A DUSP5 myc-expressing cells at all times. *B*, similar ANOVAs of MI measures revealed a significant difference between WT DUSP5-myc and R53A/R54A DUSP5-myc expressing cells (*p* < 0.01), and post hoc Bonferroni tests revealed significant differences at all time points (*, *p* < 0.05; **, *p* < 0.01). One way ANOVAs of the myc data (*C*) revealed time as a significant source of variation, and post hoc Dunnett's tests revealed significant increases at 30 and 60 min (compared with 0 min) for each construct.

As an alternative approach we increased feedback using an ERK-responsive Egr1 promoter to express DUSP5 a nuclear-inducible and ERK-specific DUSP (36, 37). The promoter was also used to express R53A/R54A DUSP5, where the kinase interacting motif is mutated to inhibit binding to and inactivation of ERK (30, 36). In embryonic fibroblasts transduced with Ad for Egr1-driven DUSP5 myc expression (Ad DUSP5 myc), ERK activation increased DUSP5 and thereby increased ERK-mediated feedback (30). We varied Ad DUSP5 myc titer and as shown (Fig. 6*A* and *B*), 10^−7^ m GnRH caused a time- and Ad titer-dependent increase in WT DUSP5 myc and R53A/R54A DUSP5 myc levels in GnRHR-expressing HeLa cells. Myc levels were similar for both constructs (at matched titers and times). Most importantly, WT DUSP5 myc reduced the GnRH effect on ppERK, whereas R53A/R54A DUSP5 myc did not (Fig. 6, *C* and *D*), validating this strategy for increasing ERK-mediated feedback. We next constructed concentration-response curves for GnRH effects on ppERK with a smaller range of time points and with both Ad at 4 pfu/nl (Fig. 7). GnRH caused the expected concentration-dependent and transient increase in ppERK in cells expressing R53A/R54A DUSP5, and the population-averaged responses were reduced by Egr1-driven expression of WT DUSP5 (Fig. 7*A*). Moreover, I(ppERK;GnRH) was lower in the cells with WT DUSP5 (Fig. 7*B*). Interestingly, ppERK levels and I(ppERK:GnRH) were reduced by WT DUSP5 after only 5 min with GnRH despite the fact that the GnRH effect on DUSP5 myc expression was not evident until 30 min (Fig. 7*C*). However, there was a small increase in DUSP5 myc before GnRH stimulation (∼20 AFU above basal at time 0; Fig. 7*C*), presumably because basal ERK activity causes Egr1-driven DUSP expression in the ∼16 h between transduction and stimulation. Accordingly, although this initial effect is likely due to negative feedback on ERK signaling, it could well include signaling before GnRH addition. Nevertheless, having established that reducing ERK-mediated negative feedback can reduce the reliability of GnRH sensing (Fig. 5), we now show that increasing it can also do so (Fig. 7).

##### The Reliability of GnRH Sensing Is Influenced by Protein Neosynthesis

**FIGURE 8.**
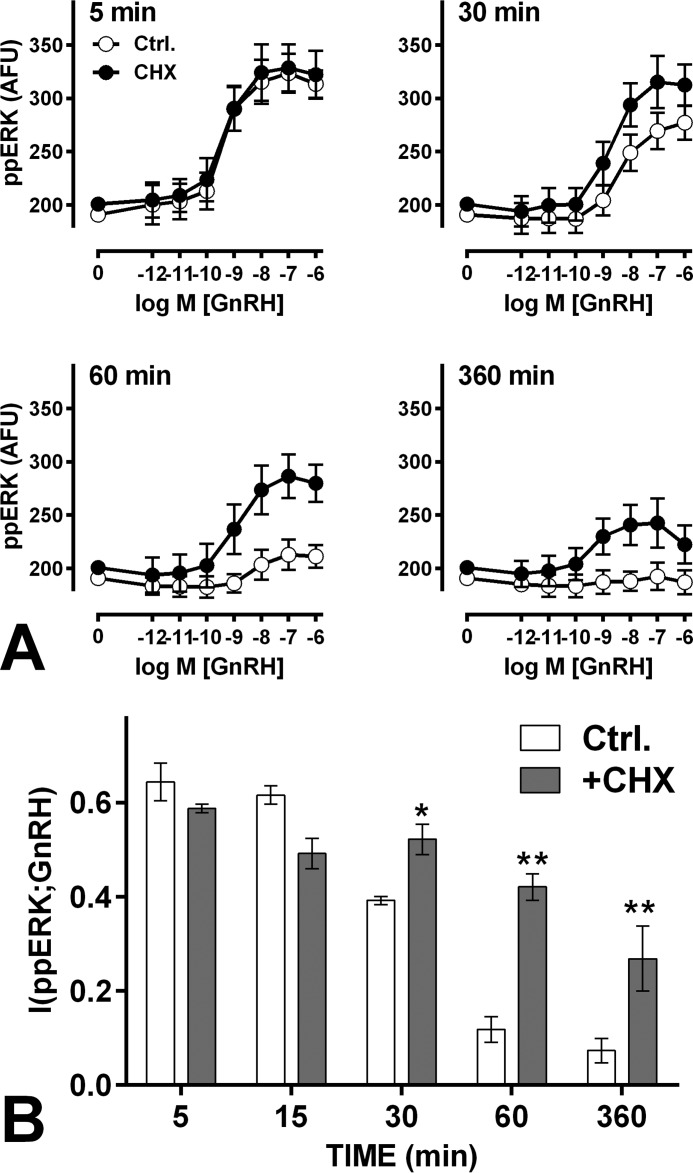
**Cycloheximide can improve GnRH sensing.** *Panel A*, cells were treated as in Fig. 1, except that they were preincubated for 30 min in control medium (*filled symbols*) or with 30 μm CHX (*open symbols*) before GnRH stimulation. Single cell data were used to calculate I(ppERK;GnRH) as shown in *panel B*. 15-min data are omitted from *panel A* for clarity. *A*, two-way ANOVAs of the ppERK2 data revealed significant differences between control and CHX-treated cells at 30, 60, and 360 min. *B*, similar ANOVAs of the MI measures revealed a significant CHX effect, and post hoc Bonferroni tests revealed significant differences between control and CHX-treated cells 30, 60, and 360 min (*, *p* < 0.05; **, *p* < 0.01).

ERK-activating stimuli characteristically increase nuclear-inducible DUSP expression, and GnRH does so in the HeLa cell model used here (20). To test whether protein neosynthesis also influences GnRH sensing, we used the protein synthesis inhibitor cycloheximide (CHX). GnRH caused the expected time- and dose-dependent increase in population-averaged ppERK levels (Fig. 8). CHX did not influence the initial response (Fig. 8*A*, 5-min data), but at later time points it increased maximal responses to GnRH. This is consistent with earlier work (20) and implies that protein neosynthesis contributes to the transient nature of GnRH effect, because it is needed for GnRH-stimulated expression of nuclear-inducible DUSPs. Most importantly, the more sustained effect of GnRH on ppERK levels was associated with a more sustained increase in GnRH sensing, as indicated by the fact that I(ppERK;GnRH) values were unaltered by CHX at 5 and 15 but were increased by CHX with 30, 60, and 360 min of GnRH stimulation (Fig. 8*B*). Interestingly, the initial effects of WT DUSP expression on ppERK and sensing (5 min data in Figs. 7, *A* and *B*) were not prevented by CHX (not shown), confirming that they cannot be attributed to induction of DUSP5 expression by GnRH in this time frame.

##### Relationships between GnRHR Structure and GnRHR-mediated Information Transfer

**FIGURE 9.**
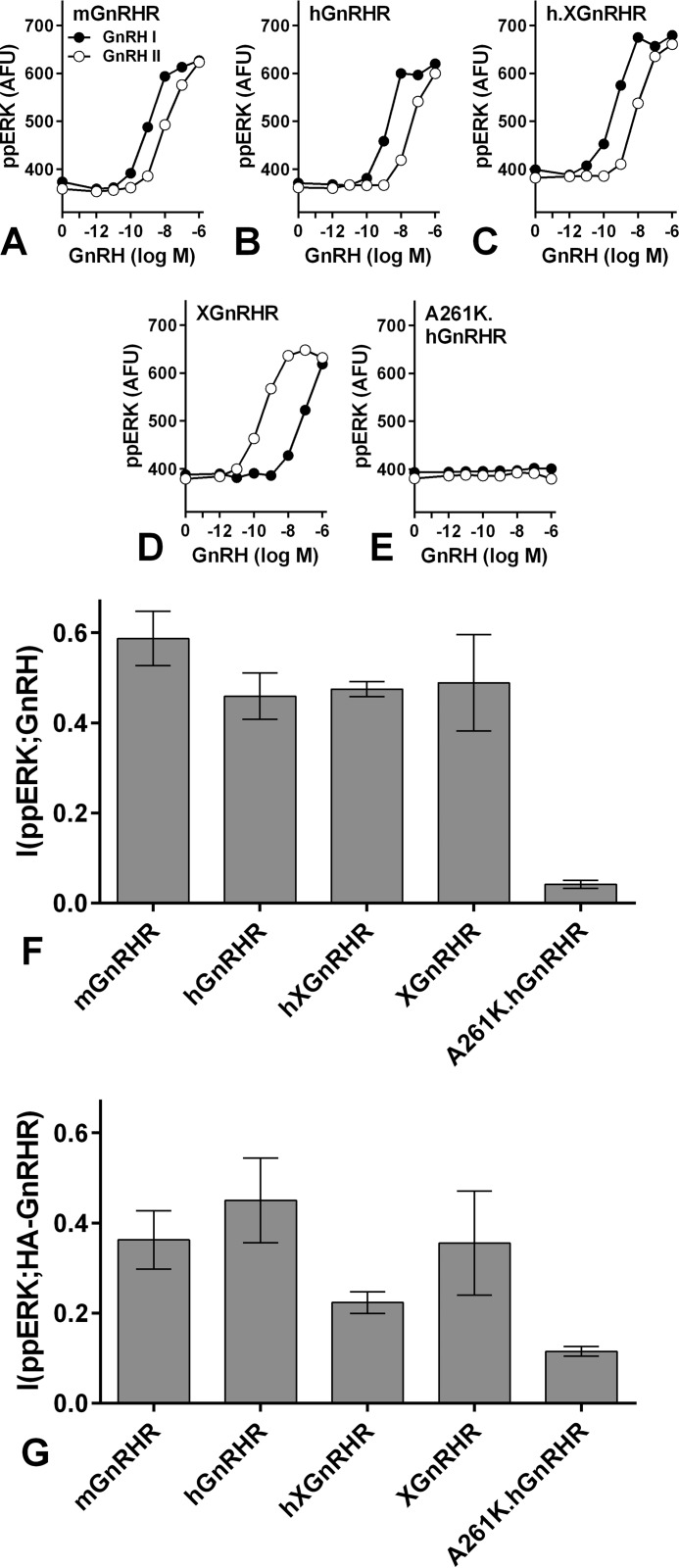
**GnRH sensing via different types of GnRHR and ERK.** *A–E*, cells grown in 6-well plates were infected with Ad to express HA -mGnRHR, -hGnRHR, -h.XGnRHR, -XGnRHR, or -A261K.hGnRHR as indicated, each at 3 titers (1, 10, and 100 pfu/nl). After 6 h they were harvested, pooled (by receptor type), transferred to 96-well plates, and cultured overnight. They were then stimulated 5 min with GnRH I or GnRH II before being processed for quantification of nuclear ppERK and whole cell HA. Data shown are the mean population-averaged ppERK values. *F*, individual cell measures were used to calculate (I(ppERK;GnRH) for each receptor using GnRH II for XGnRHR and GnRH I for all other receptors. Similar MI values were obtained when the alternative ligands were used. The same data were used to calculate the MI values shown in *panel G*, but in this case MI was calculated using individual cell measures of HA and ppERK (I(ppERK;HA-GnRHR). The data are pooled from three experiments with S.E. shown only in *panels F* and *G* for clarity. Fig. 10 shows further data pertaining to the analysis in *panel G*. One way ANOVAs (for the signaling receptors) revealed that receptor type is not a significant source of variation for *panels F* and *G*.

Having found that downstream (ERK-mediated) feedback influences information transfer via GnRHR, we assessed whether upstream (ERK-independent) feedback would also do so. We exploited the fact that most GnRHR rapidly desensitize, whereas type I mammalian GnRHR do not (17, 23, 24). Accordingly, we compared HeLa cells transduced with Ad to express HA-tagged hGnRHR and mGnRHR (type I mammalian GnRHR lacking COOH-terminal tails) or XGnRHR (which have COOH tails). We also included a signal dead point mutant of the hGnRHR (A261K.hGnRHR) and a previously characterized chimera, the entire hGnRHR with the COOH-terminal tail of the XGnRHR added (h.XGnRHR; Ref. 29). Cells were stimulated for 5 min with GnRH I (also known simply as GnRH) or with GnRH II, and as expected, GnRH I was more potent than GnRH II at mGnRHR, hGnRHR, and h.XGnRHR, whereas GnRH II was more potent at XGnRHR, and neither increased ppERK via A261K.hGnRHR (Fig. 9, *A–E*). MI values were calculated for GnRH II at XGnRHR and GnRH I at the others. I(ppERK;GnRH) was negligible for A261K.hGnRHR. I(ppERK;GnRH) values were 0.4–0.6 for each of the other GnRHR (Fig. 9*F*) and did not differ significantly (*p* > 0.05). Thus, the absence or presence of COOH tail and the associated occurrence or lack of rapid receptor desensitization did not influence GnRH sensing.

**FIGURE 10.**
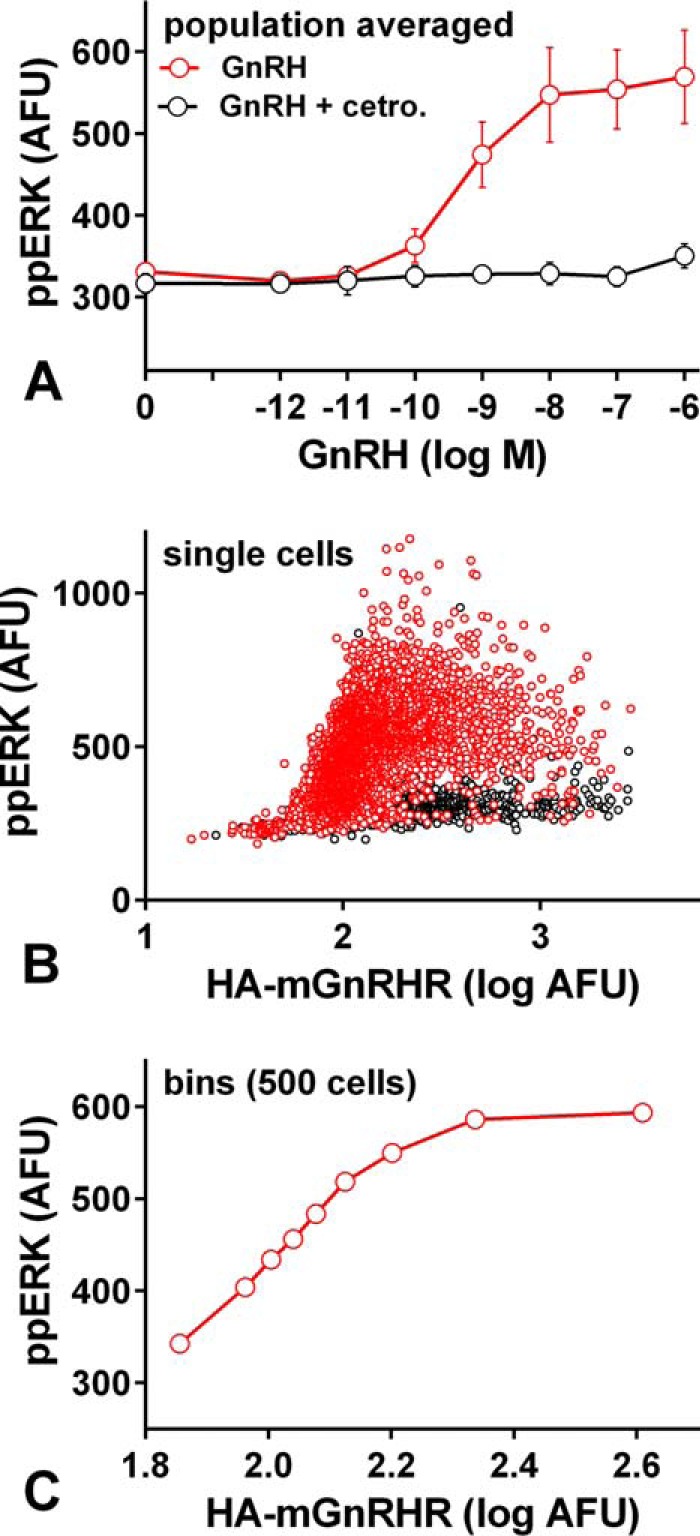
**Quantifying MI between HA-tagged mGnRHR expression and ppERK.** *Panel A*, cells grown in 6-well plates were infected with Ad for HA-tagged mGnRHR, at 3 titers (1, 10 and 100 pfu/nl). After 6 h they were pooled and transferred into 96-well plates for overnight culture. They were then stimulated 5 min with indicated GnRH concentration with or without 10^−7^ m Cetrorelix (as indicated) before being fixed, stained, imaged, and analyzed for nuclear ppERK and whole cell HA. Data shown are the population-averaged ppERK values pooled from three experiments. *Panel B* shows single cell data (nuclear ppERK plotted against log_10_ HA) from one such experiment and only for cells stimulated with 10^−7^ m GnRH without (*red circles*) or with (*black circles*) 10^−7^ m Cetrorelix. The 10^−7^ m GnRH-stimulated cells were also ranked and sorted into bins of 500 cells with increasing HA. *Panel C* shows ppERK plotted against HA for each of these bins (means ± S.E., *n* = 500 cells). Note that increasing GnRH concentration increases the ppERK response at fixed receptor number (*panel A*) and that increasing receptor number increases ppERK at fixed GnRH concentration (*panel C*). MI can then be calculated for both of these input-output pairs as shown in Fig. 9, *F* and *G*.

**FIGURE 11.**
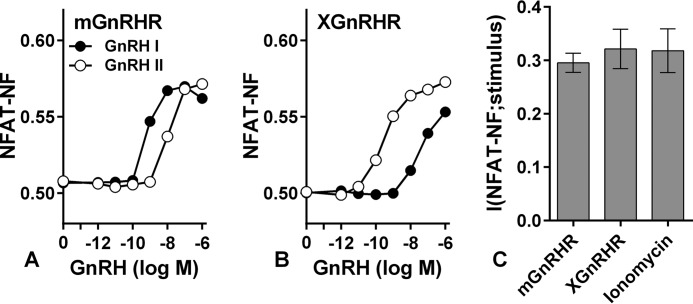
**GnRH sensing via different types of GnRHR and NFAT.** *A* and *B*, cells grown in 6-well plates were infected with Ad to express NFAT1c-EFP along with HA-mGnRHR or XGnRHR as indicated, each at 3 titers (1, 10, and 100 pfu/nl). After 6 h they were harvested, pooled (by receptor type), transferred to 96-well plates, and cultured overnight. They were then stimulated 20 min with GnRH I or GnRH II before being processed for quantification of NFAT-NF and whole cell HA. Data shown are the mean population-averaged NFAT-NF values. *C*, individual cell measures were used to calculate (I(NFAT-NF;GnRH) for each receptor using GnRH II for XGnRHR and GnRH I for mGnRHR. Similar MI values were obtained when the alternative ligands were used (not shown). In parallel experiments cells receiving no Ad GnRHR were treated with ionomycin (0 or 10^−8^ to 3 × 10^−6^ m), and I(NFAT-NF;ionomycin) was calculated from the single cell NFAT-NF measure. The data are pooled from three experiments with S.E. shown only in *panel C* for clarity.

Receptor expression levels could influence GnRH sensing (Fig. 2*B*), so as an alternative approach we calculated MI between GnRHR expression and ppERK. Fig. 10*A* shows the dose-dependent effect of GnRH on ppERK responses in HA-mGnRHR-expressing cells, where I(ppERK;GnRH) was 0.70 ± 0.04. Fig. 10*B* shows ppERK and HA levels for ∼5000 individual cells treated with 10^−7^ m GnRH. In each case data are also shown for cells treated with GnRH in the presence of the GnRHR antagonist Cetrorelix (from Merck Serono), confirming GnRHR mediation. We also binned these individual cells (10^−7^ m GnRH-stimulated cells only) according to HA expression and observed the expected positive relationship between HA-GnRHR and ppERK levels (Fig. 10*C*). Because GnRHR occupancy approaches 100% with 10^−7^ m GnRH, this is effectively a GnRHR occupancy-response relationship, and the individual cell data underpinning it can be used to calculate MI between HA expression (occupancy) and ppERK responses (I(ppERK;HA-GnRHR)) as an alternative measure for receptor comparison. Re-analyzing the data from Fig. 9, *A–E*, we found that I(ppERK;HA-GnRHR) values are lower than I(ppERK;GnRH) values (compare Fig. 9, *F* and *G*). Nevertheless, I(ppERK;HA-GnRHR) values were comparable for the four signaling receptors, re-enforcing the conclusion that the absence or presence of rapid receptor desensitization does not influence GnRH sensing in this model. In a final series of experiments we also determined MI for GnRH signaling to NFAT in HeLa cells transduced with Ad mGnRHR or Ad XGnRHR along with Ad NFAT1c-EFP. GnRH I and II caused concentration-dependent translocation of NFAT to the nucleus with the expected difference in potency (GnRH I being more potent at mGnRHR and less potent at XGnRHR) (Fig. 11). I(NFAT-NF;GnRH) values calculated from the single cell data were similar for the two receptors (Fig. 11*C*). In addition, concentration-response curves were constructed for ionomycin, and MI for this receptor-independent stimulus was comparable to that with either receptor (Fig. 11*C*).

## Discussion

Mutual Information (MI) is a statistical measure of the quality of the inference (or prediction) of the signal from the response (1) that can be used to measure information transfer via cell signaling pathways and networks. Here we do so for GnRHR, G-protein-coupled receptors that engage a bifurcating system in which PLC activation mediates activation of Ca^2+^ effectors (including NFAT) as well as PKCs and their effectors (including ERK). From single cell measures of ERK activation, we estimate information transfer (I(ppERK;GnRH)) to be <1 bit in HeLa cells transduced with Ad GnRHR. This is comparable with values obtained for cytokine and growth factor signaling in other systems (2, 4, 7) but is still surprisingly low for two reasons. First, we typically stimulate with eight GnRH concentrations so we deal with a 3 bit input, of which <1 bit is transferred. Second, population-averaged outputs are graded over a wide range of GnRH concentrations (Fig. 1*A*) yet an MI of <1 implies that single cells are unable to unambiguously distinguish between just two (equally probable) inputs (*i.e.* with and without GnRH). We suspected that this could be due to use of a heterologous expression system, but this is not to be the case as I(ppERK;GnRH) values were similar in HeLa cells (exogenous GnRHR) and LβT2 gonadotropes (endogenous GnRHR). Another possibility is that other pathways sense GnRH more reliably, but when information transfer to NFAT was estimated from single cell measures of the nuclear fraction of NFAT1c-EFP, I(NFAT-NF;GnRH), values were <0.5 bits in both cell models.

In both cell types GnRHR expression could influence sensing, and we found that increasing GnRHR number (by varying Ad GnRHR) caused a corresponding increase in I(ppERK;GnRH) (Fig. 2*B*). Rodent gonadotropes and gonadotrope cell lines express ∼25–75,000 GnRHR per cell (13, 29, 34, 35), and sensing increased as GnRHR number was increased through this range but was not further increased by expression at ∼180,000 sites/cell (Fig. 2*B*). Physiologically, GnRHR expression is tightly controlled with increases through puberty, the oestrous cycle, and lactation (13, 34), so our data imply that such changes could well influence information transfer via GnRHR. They also imply that we do not underestimate sensing by over- or under-expressing GnRHR in the HeLa cell model. Indeed, it is noteworthy that maximal I(ppERK;GnRH) and I(ppERK;PDBu) values were similar just as maximal I(NFAT-NF;GnRH) and I(NFAT-NF;ionomycin) were similar (Figs. 1 and 11), implying that features other than GnRHR occupancy limit information transfer to ERK and NFAT in these cells.

Another possibility is that single time point measures underestimate information transfer as expected when cells infer inputs (*i.e.* GnRH concentrations) from trajectories of outputs (*i.e.* ppERK levels) over time (8). The time-courses reveal, for example, that I(ppERK;GnRH) is higher at 5 than at 360 min (Fig. 1), but this clearly does not mean that a cell obtains less information over 360 min than it had over 5 min. Instead, this shows that the 360-min snapshot underestimates information transferred over the 360 min. Measuring MI for ERK-driven transcription is an alternative approach that could be sensitive to ppERK trajectory, and consistent with this, we found that I(Egr1;PDBu) was greater than I(Egr1;GnRH) in HeLa cells, presumably because PDBu has a more sustained effect than GnRH on ppERK and causes a more marked increase in Egr1-driven zsGREEN expression (Fig. 1). Similarly, I(Egr1;GnRH) was greater in LβT2 cells, presumably because GnRH has a more sustained effect on ppERK and causes a more pronounced increase in Egr1-driven zsGREEN expression than in HeLa cells (Fig. 2 and data not shown). Thus the system senses sustained stimulation more reliably and must, therefore, be sensitive to ERK activation trajectory.

Having established that GnRH sensing by single cells is relatively unreliable, we explored mechanisms that could mitigate information loss. We focused on negative feedback, which has the potential to cause desensitization (*i.e.* to reduce average system output despite a constant input) and to reduce variability, effects that would tend to harm or protect sensing, respectively. ERK responses are modulated by multiple feedback loops (9, 10), so to explore the effects on sensing we developed a stochastic model with a slow feedback loop (paralleling a transcription-dependent increase in DUSP) and a fast feedback loop (paralleling ERK-mediated inhibitory phosphorylation of Raf). Negative feedback can preserve sensing by opposing the “leak” due to basal activity in a generic protein phosphorylation and dephosphorylation pathway (4), so we include basal activity in the current model. Simulations (Figs. 3 and 4) revealed that the negative feedback loops can both increase or reduce I(ppERK;S), because for both, strong negative feedback impairs sensing by reducing the output dynamic range, whereas intermediate strength feedback can improve it by opposing noise and/or leak. The simulations show bell-shaped relationships between fast feedback strength and sensing that are most obvious with little or no slow feedback (*blue lines* in Fig. 4, *A–D*) and between slow feedback strength and sensing that are most evident when fast feedback is weak or intermediate (Fig. 4, *E* and *F*). They also show a pronounced effect of slow feedback after only 5 min when fast feedback is low (Fig. 4*A*), because this feedback is activated by and opposes the effect of leak during the pre-equilibration period.

**FIGURE 12.**
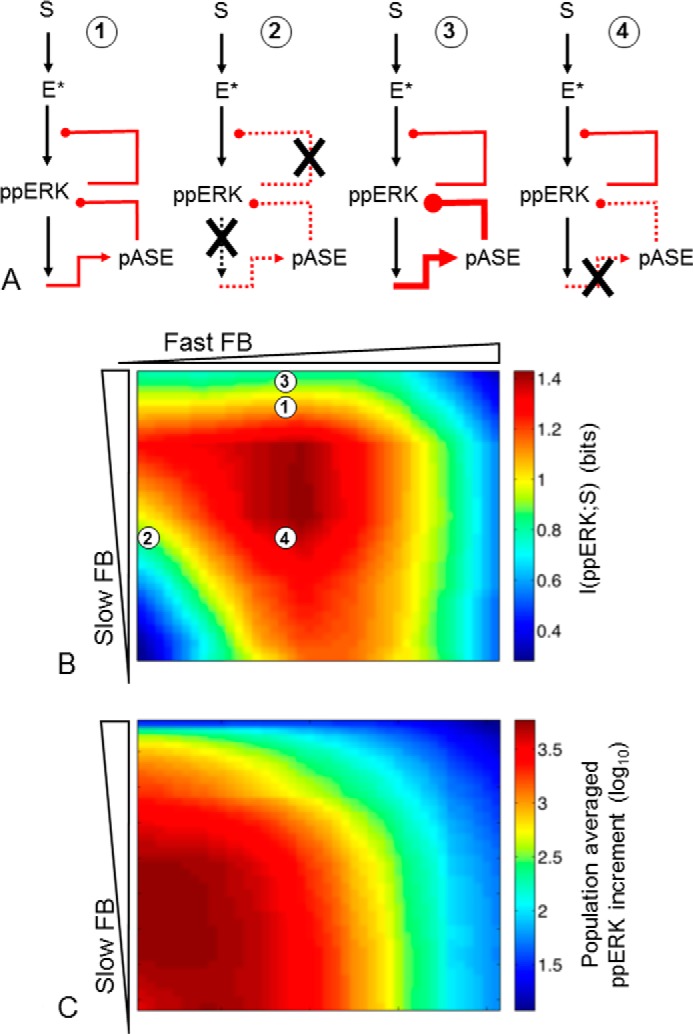
**ERK data summary.** *Panel A*, simplified model topologies for control cells (*1*), cells with catalytically inactive ERK (*2*), and cells where the slow (transcription-dependent) pathway is increased (*3*) or reduced (*4*). *Panel B*, heat map of predicted I(ppERK;S) values for varied fast and slow feedback (*FB*), generated from the data set shown (in part) in Figs. 2 and 3, with fast FB and slow FB at of −0.5–3.5 and 0–4 (log_10_ scale), respectively. *Panel C*, heat map of population-averaged S-stimulated ppERK responses (maximum-minimum) obtained by fitting simulation single cell responses shown (in part) in Fig. 2, with the same FB ranges as *A*. The *numbers 1–4* on B correspond to the schematics, showing possible positions of these topologies in the parameter space and illustrating consistency between the simulations and experimental manipulations.

The features described above are illustrated in Fig. 12, where *panel B* shows I(ppERK;S) at different feedback levels and *C* shows the maximal substrate-driven increase in ppERK. The difference between these heat maps provides a striking demonstration of the fact that changes in population-averaged responses (*panel C*) do not necessarily parallel changes in information transfer (*panel B*). Indeed, the overriding effect of increasing fast or slow feedback is to reduce population-averaged ppERK values, whereas for both feedbacks I(ppERK;S) is maximal at intermediate levels. We also illustrate our experimental manipulations in schematic form (Fig. 12*A*) with *1* representing the normal condition where fast and slow feedback pathways are intact. In cells expressing catalytically inactive K52R-ERK2 (Fig. 5), both feedback paths are broken (*2*), and I(ppERK;S) is reduced, moving from *1* to *2* in Fig. 12*B*. In cells expressing the construct for Egr1-driven expression of WT DUSP5 (Fig. 7) slow feedback is increased (*3*), and this reduces I(ppERK;S), moving from *1* to *3* in Fig. 12*B*. Finally, when cells are stimulated in the presence of cycloheximide (Fig. 8) the slow feedback loop is broken (*4*), and this increases I(ppERK;S), moving from *1* to *4* in Fig. 12*B*. Clearly there are a number of caveats to consider here, notably the fact that we have not shown the cycloheximide effect to be due to prevention of DUSP neosynthesis, and we do not consider other proteins for which expression could be directly or indirectly influenced by cycloheximide. Nevertheless, the behaviors outlined above are entirely consistent with both the stochastic modeling and the wet laboratory data.

Rapid homologous desensitization is an upstream adaptation that uncouples GPCRs from their G-proteins and has the potential to modulate ERK signaling, so we also compared mammalian GnRHR (hGnRHR and mGnRHR) that do not desensitize and non-mammalian GnRHR (XGnRHR) that do (23). hGnRHR and mGnRHR are selectively activated by GnRH I, whereas the XGnRHR is selective for GnRH II (GnRH and GnRH I are identical; we use the numeral here to distinguish GnRH I from II), and population-averaged data revealed the expected dose-dependent increases in ppERK for each receptor (Fig. 9). The h.XGnRHR chimera mediated GnRH I-selective ERK activation, and the signal dead A261K.hGnRHR did not. Most importantly, I(ppERK;GnRH) values were indistinguishable for the functional GnRHR (Fig. 9) irrespective of whether or not they rapidly desensitize.

Because GnRHR number influences sensing (Fig. 2), differences in receptor number could influence the work outlined above, but the GnRHR used were HA-tagged so we addressed this in two ways. First, we measured population-averaged HA levels and found them to be indistinguishable (*p* > 0.05 by ANOVA) for all five GnRHRs. Second, we used the measured HA levels as inputs for MI calculation. In GnRH-stimulated cells there were positive relationships between HA and ppERK levels (mGnRHR data in Fig. 10), and the single cell measures were also used to calculate MI between GnRHR occupancy (*i.e.* HA level) and ppERK (Figs. 9 and 10). This again revealed comparable information transfer for each of the active GnRHR (and negligible MI for the signal dead A261K.hGnRHR). Thus, we show that for these active GnRHR, I(ppERK;GnRH) values are indistinguishable under conditions of comparable receptor expression (Fig. 9*F*), and I(ppERK;HA-GnRHR) values are also comparable (Fig. 9*G*). An interesting finding is that I(ppERK;HA-GnRHR) was lower than I(ppERK;GnRH) (compare Fig. 9 *panels F* and *G*, taking HA-mGnRHR for example). We had anticipated the opposite, that variation in GnRHR expression would reduce I(ppERK;GnRH) and that we would be able to correct for this by calculating I(ppERK;HA-GnRHR). Instead, it appears that the additional experimental measure (HA is measured for calculation of I(ppERK;HA-GnRHR), whereas GnRH concentration is fixed for calculation of I(ppERK;GnRH)) introduces sufficient experimental error variation to actually reduce MI. This does not alter the conclusion but highlights the fact that MI estimates are inevitably dependent on the experimental methods. In a final series of experiments we found that I(NFAT-NF;GnRH) values were similar for effector engagement via mGnRHR or XGnRHR (Fig. 11). It remains possible that these receptors transfer information differently to other system outputs or to the same output under different conditions (*i.e.* on more sustained activation), and we clearly cannot extrapolate these findings to other GPCRs. Nevertheless, our overriding observation is that information transfer via GnRHR was not measurably influenced by the occurrence or absence of rapid receptor desensitization, and this stands in contrast to the clear influence of downstream adaptive processes on GnRH sensing.

In summary, we used single cell measures to quantify information transfer via GnRHR to ERK and NFAT, and under all tested conditions, MI was <1 Bit, implying that these pathways do not enable cells to distinguish even two (equally probable) states of the environment. Focusing on ERK signaling, our stochastic modeling revealed that fast and slow negative feedback loops both have the potential to protect or harm sensing. These predictions were confirmed for the GnRHR/ERK pathway by exploring the effects of transcription-dependent and transcription-independent negative feedback loops on ERK activation. However, comparison of GnRHR that do or do not desensitize revealed no evidence that rapid receptor desensitization influences GnRH sensing. Population-averaged measures support the conventional wisdom that negative feedback causes desensitization, but we find that hormone sensing is actually most reliable at intermediate feedback levels. Because such feedback reduces the population-averaged system outputs, hormone sensing was optimal with intermediate feedback and submaximal population-averaged responses.

## Author Contributions

K. L. G., R. M. P., and M. V. contributed equally to this work. C. A. M. conceived and coordinated the study and wrote the first draft of the paper. M. V. and C. B. planned and performed the stochastic modeling. The remaining experiments were designed, performed, and analyzed by K. L. G., R. M. P., C. J. C., T. P., G. R. P., and K. T.-A. All authors reviewed the results and approved the final version of the manuscript.

## Footnotes

3 The abbreviations used are:

MI: mutual information (also I(Z;S), the mutual information between signal (*S*) and response (*Z*))
GnRH: gonadotropin-releasing hormone (GnRH is also known as GnRH l; we use the numeral only when necessary to distinguish from GnRH II)
GnRHR: GnRH receptor (also hGnRHR, mGnRHR, XGnRHR for human, mouse, and *X. laevis* GnRHR, respectively)
PDBu: phorbol 12,13-dibutyrate
ppERK: dual phosphorylated (activated) ERK
DUSP: dual specificity phosphatase
NFAT: nuclear-factor of activated T-cells
GPCR: G-protein-coupled receptor
Ad: adenovirus
CHX: cycloheximide
AFU: arbitrary fluorescence units
RE: response element
ANOVA: analysis of variance.
